# The mitogenome of *Halotydeus destructor* (Tucker) and its relationships with other trombidiform mites as inferred from nucleotide sequences and gene arrangements

**DOI:** 10.1002/ece3.8133

**Published:** 2021-09-22

**Authors:** Joshua A. Thia, Neil D. Young, Pasi K. Korhnen, Qiong Yang, Robin B. Gasser, Paul A. Umina, Ary A. Hoffmann

**Affiliations:** ^1^ Bio 21 Institute, School of BioSciences The University of Melbourne Melbourne Victoria Australia; ^2^ Department of Veterinary Biosciences, Melbourne Veterinary School The University of Melbourne Melbourne Victoria Australia; ^3^ Cesar Australia Brunswick Victoria Australia

**Keywords:** Bayesian inference, *Halotydeus destructor*, mitochondrial DNA, Penthaleidae, phylogenetics, redlegged earth mite, Trombidiformes

## Abstract

The redlegged earth mite, *Halotydeus destructor* (Tucker, 1925: Trombidiformes, Eupodoidea, Penthaleidae), is an invasive mite species. In Australia, this mite has become a pest of winter pastures and grain crops. We report the complete mitogenome for *H. destructor*, the first to represent the family Penthaleidae, superfamily Eupodoidea. The mitogenome of *H. destructor* is 14,691 bp in size, and has a GC content of 27.87%, 13 protein‐coding genes, two rRNA genes, and 22 tRNA genes. We explored evolutionary relationships of *H. destructor* with other members of the Trombidiformes using phylogenetic analyses of nucleotide sequences and the order of protein‐coding and rRNA genes. We found strong, consistent support for the superfamily Tydeoidea being the sister taxon to the superfamily Eupodoidea based on nucleotide sequences and gene arrangements. Moreover, the gene arrangements of Eupodoidea and Tydeoidea are not only identical to each other but also identical to that of the hypothesized arthropod ancestor, showing a high level of conservatism in the mitogenomic structure of these mite superfamilies. Our study illustrates the utility of gene arrangements for providing complementary information to nucleotide sequences with respect to inferring the evolutionary relationships of species within the order Trombidiformes. The mitogenome of *H. destructor* provides a valuable resource for further population genetic studies of this important agricultural pest. Given the co‐occurrence of closely related, morphologically similar Penthaleidae mites with *H. destructor* in the field, a complete mitogenome provides new opportunities to develop metabarcoding tools to study mite diversity in agro‐ecosystems. Moreover, the *H. destructor* mitogenome fills an important taxonomic gap that will facilitate further study of trombidiform mite evolution.

## Graphical abstract

We report the complete mitogenome sequence of the redlegged earth mite, Halotydeus destructor (Tucker). We used Bayesian phylogenetic analyses to contextualize the relationship of H. destructor with other trombidiform mites. Phylogenies were inferred from protein‐coding and rRNA genes using nucleotide sequences and a novel characterization of gene order arrangements.

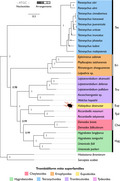

## 1 INTRODUCTION

The redlegged earth mite, *Halotydeus destructor* (Tucker, 1925), belongs to the order Trombidiformes (superfamily Eupodoidea; family Penthaleidae). *Halotydeus destructor* is an important pest in Australian agriculture, attacking winter pasture, grain crops, and oilseed crops in temperate southern cropping regions (Ridsdill‐Smith, 1997). This mite species was introduced into Australia from South Africa in the early 1920s (Newman, 1925). Since its establishment, this mite has been mostly controlled using pesticide, with a long history of organophosphate and pyrethroid chemical usage (Ridsdill‐Smith et al., 2008). However, pyrethroid resistance has evolved and become widespread (Arthur et al., 2021; Edwards et al., 2018; Yang et al., 2020), and more recently, geographically isolated cases of organophosphate resistance have been identified (Arthur et al., 2021; Umina et al., 2017).

Trombidiform mites represent a diverse group that includes other agricultural pests such as spider mites and gall mites, as well as mites that are medically important, such as chiggers. The order Trombidiformes includes more than 25,000 species and over 2,000 genera that have been assigned to 151 families and two suborders (Zhang et al., 2011). Within both suborders, there are at least seven independent parallel evolutionary events leading to herbivory arising from a parasitic ancestral state (Lindquist, 1998). The superfamily Eupodoidea within the Trombidiformes has ~340 species, which are mostly mites associated with terrestrial soil environments (Qin, 1996). The family Penthaleidae (Oudemans, 1931) includes five genera, which represent global pests in the genus *Penthaleus* (Umina et al., 2004) and five recognized species of *Halotydeus* (Qin & Halliday, 1996).

Despite their importance as pests, genomic resources for the Penthaleidae are poorly developed. Such resources would significantly assist studies of population demography, pesticide resistance, and phylogenetic relationships both within the family and within the context of the superfamily Eupodoidea, where relationships remain unclear (Szudarek‐Trepto et al., 2020). In particular, complete mitochondrial sequences could provide greater capacity to resolve taxonomy among members of the Trombidiformes, given the unusually high incidence of mitogenomic rearrangements that have occurred in this order that complicate phylogenetic analyses of sequence data from single genes (Edwards et al., 2011; Palopoli et al., 2014; Shao et al., 2005). Structural haplotypes in mitochondrial gene arrangements have provided insights into evolutionary relationships across diverse taxa (Froufe et al., 2020; Gong et al., 2019; Inoue et al., 2003; Kutyumov et al., 2021), but to date, such analyses have been relatively small in scope with respect to the Trombidiformes, typically focusing on a few species (Edwards et al., 2011; Li & Xue, 2019; Palopoli et al., 2014; Shao et al., 2005; Xue et al., 2017; Xue et al., 2016).

Here, we present the complete mitogenome of *H. destructor*, the first to be sequenced from a mite in the superfamily Eupodoidea. We procured complete mitogenomes from all other species of the Trombidiformes available publicly to link the evolutionary relationships of *H. destructor* with mites in this order. In addition to standard phylogenetic analyses using nucleotide sequences from mitochondrial genes, we also analyzed gene arrangements. We show that structural haplotypes of protein‐coding and rRNA genes can provide complementary information to nucleotide sequences for elucidating the evolutionary relationships within this diverse order of mites.

## 2 METHODS

Samples of adult *H. destructor* were collected from recreational pasture in Wantirna South, Melbourne, VIC, Australia (S37°52′11.6″ E145°11′46.1″), in June 2020. Collection was made via suction using a blower vacuum with a fine gauze mesh placed over the end of the vacuum tube. Mites were placed into a plastic container with paper towel and vegetation for transportation. Once in the laboratory, *H. destructor* individuals were identified and separated from other invertebrates. Although distinguishing sibling species in the genus *Halotydeus* by morphology is challenging, *H. destructor* is the only member from this genus present in Australia based on previous population genetic assessments of this species across its native and invasive range with different marker systems (Hill et al., 2016; Weeks et al., 1995; Yang et al., 2020).

DNA was extracted from pooled individuals (*n* = 70) using a DNeasy^®^ Blood & Tissue Kit (Qiagen, Hilden, Germany). A continuous long‐read library was prepared and sequenced using PacBio Sequel II technology by Berry Genomics (Berry Genomics Co. Ltd, Beijing, China). The PacBio reads were parsed with the long‐read assembler canu v2.0 (Koren et al., 2017) using the “correction” method. The corrected reads were then subset using seqtk v1.3‐r106 (Li, 2019), setting a minimum read length of 5,000 bp. We assembled the corrected reads using flye v2.8 (Kolmogorov et al., 2019) and extracted circular contigs. Using bwa mem (Li & Durbin, 2009), we mapped the *cox*1 sequence from the parasitic mite, *Demodex brevis* (GenBank accession KM114225: position 1–1536), to these circular contigs with relaxed parameters: –A1 –B1 –O2. Using samtools v1.9 (Li et al., 2009), we identified one contig that mapped to the *D. brevis cox*1 sequence. This contig was 29,383 bp long and contained multiple copies of the mitogenome merged into a single sequence. By annotating this contig, we defined a single copy mitogenome sequence for *H. destructor*.

The protein‐coding, rRNA, tRNA genes, and hypothetical replication origins within the *H. destructor* mitogenome were annotated using a combination of mitos2 v2.0.8 (Bernt et al., 2013), arwen (Laslett & Canbäck, 2008), and geneious prime v2021.1.1 (Geneious, 2021). Initially, mitos2 was used to infer protein‐coding, rRNA, and tRNA genes from the long‐read contig. The 29,383 bp long contig was trimmed and repositioned to start at the first annotated *cox*1 gene and end at the next proceeding *cox*1 annotation. Thus, the initial long‐read contig was reduced to a single copy of the mitogenome (14,691 bp long). The tRNA‐Val gene was additionally annotated using arwen. We used the live annotation feature of geneious prime to validate the positions of the rRNA genes using sequences from all sampled trombidiform mites (Table 1).

**TABLE 1:** List of mite species, their taxonomy, their taxonomic authorities, and the NCBI accessions for their mitogenomes

Species	Infraorder	Superfamily	Family	Authority	Accession	Reference^a^
*Demodex brevis*	Eleutherengona	Cheyletoidea	Demodicidae	Akbulatova (1963)	KM114225	Palopoli et al. (2014)
*Demodex folliculorum*	Eleutherengona	Cheyletoidea	Demodicidae	Simon (1842)	KM114226	Palopoli et al. (2014)
*Epitrimerus sabinae*	Eupodina	Eriophyoidea	Eriophyidae	Xue & Hong (2005)	NC_029208	Xue et al. (2016)
*Leipothrix* sp.	Eupodina	Eriophyoidea	Eriophyidae	Keifer (1966)	KX027362	Xue et al. (2017)
*Phyllocoptes taishanensis*	Eupodina	Eriophyoidea	Eriophyidae	Xue & Hong (2005)	KR604967	Xue et al. (2016)
*Rhinotergum shaoguanense*	Eupodina	Eriophyoidea	Diptilomiopidae	Xue, Song & Hong (2009)	NC_034150	Xue et al. (2017)
*Hygrobates longiporus*	Anystina	Hygrobatoidea	Hygrobatidae	Thor (1898)	LC552026	Hiruta et al. (2020)
*Hygrobates taniguchii*	Anystina	Hygrobatoidea	Hygrobatidae	Imamura (1954)	LC552027	Hiruta et al. (2020)
*Unionicola foili*	Anystina	Hygrobatoidea	Unionicolidae	Edwards & Vidrine (1995)	EU856396	Ernsting et al. (2009)
*Unionicola parkeri*	Anystina	Hygrobatoidea	Unionicolidae	Vidrine (1987)	HQ386015	Edwards et al. (2011)
*Panonychus citri*	Eleutherengona	Tetranychoidea	Tetranychidae	McGregor (1916)	HM189212	Yuan et al. (2010)
*Panonychus ulmi*	Eleutherengona	Tetranychoidea	Tetranychidae	Koch (1836)	NC_012571	
*Tetranychus cinnabarinus* ^b^	Eleutherengona	Tetranychoidea	Tetranychidae	Boudreaux (1956)	HM753535	
*Tetranychus kanzawai*	Eleutherengona	Tetranychoidea	Tetranychidae	Kishida (1927)	NC_024676	Chen et al. (2014)
*Tetranychus ludeni*	Eleutherengona	Tetranychoidea	Tetranychidae	Zacher (1913)	NC_024677	Chen et al. (2014)
*Tetranychus malaysiensis*	Eleutherengona	Tetranychoidea	Tetranychidae	Ehara (1988)	NC_024678	Chen et al. (2014)
*Tetranychus phaselus*	Eleutherengona	Tetranychoidea	Tetranychidae	Ehara (1960)	NC_024679	Chen et al. (2014)
*Tetranychus pueraricola*	Eleutherengona	Tetranychoidea	Tetranychidae	Ehara & Gotoh (1996)	MG518360	Sun et al. (2018)
*Tetranychus truncatus*	Eleutherengona	Tetranychoidea	Tetranychidae	Ehara (1956)	MG518351	Sun et al. (2018)
*Tetranychus urticae*	Eleutherengona	Tetranychoidea	Tetranychidae	Koch (1836)	KJ729023	Chen et al. (2014)
*Ascoschoengastia* sp.	Anystina	Trombiculoidea	Trombiculidae	Ewing (1946)	AB300501	
*Leptotrombidium akamushi*	Anystina	Trombiculoidea	Trombiculidae	Brumpt (1910)	AB194045	
*Leptotrombidium deliense*	Anystina	Trombiculoidea	Trombiculidae	Walch (1922)	AB194044	
*Leptotrombidium pallidum*	Anystina	Trombiculoidea	Trombiculidae	Nagayo, Miyagawa, Mitamura & Tamiya (1919)	AB180098	Shao et al. (2005)
*Walchia hayashii*	Anystina	Trombiculoidea	Trombiculidae	Suzuki (1979)	AB300500	
*Riccardoella reaumuri*	Eupodina	Tydeoidea	Ereynetidae	Fain & van Goethem (1986)	LC601993	
*Riccardoella tokyoensis*	Eupodina	Tydeoidea	Ereynetidae	Waki & Shimano (2018)	LC601992	
*Histiostoma feroniarum*	Acaridia	Histiostomatoidea	Histiostomatidae	Dufour (1839)	MF596167	Xue et al. (2018)
*Sarcoptes scabiei*	Psoroptidia	Sarcoptoidea	Sarcoptidae	De Geer (1778)	NC_031334	Mofiz et al. (2016)

Note

Shading groups trombidiform mites from the same superfamily and groups the sarcoptiform outgroup taxa from the trombidiform mites.

^a^ References are indicated where GenBank accessions could be attributed to published studies.

^b^ *Tetranychus cinnabarinus* is believed to be synonymous with *Tetranychus urticae* (Auger et al., 2013).

[Correction added on 28 September 2021 after first online publication: Table note has been updated in this version.]

To assess the location of the putative control region in the *H. destructor* mitogenome, we examined the position of the mitos2‐inferred replication origins. The control region is expected to occur in a mitogenomic region devoid of coding potential and of low GC content (Zhang & Hewitt, 1997). To estimate GC content, we implemented a sliding window approach (100 bp with 50 bp overlap) in R v4.0.2 using a custom script. We then visually inspected the regions of the *H. destructor* mitogenome where replication origins, predicted with mitos2, coincided with coding gaps and low GC content.

To contextualize the evolutionary relationship of the mitogenome of *H. destructor* with other trombidiform mites, we inferred phylogenetic trees from nucleotide sequences of mitochondrial protein‐coding and rRNA genes, as well as their gene arrangements within the mitogenome. We downloaded mitogenomes from 27 species of Trombidiformes, and two species of Sarcoptiformes that were used as out‐groups, from GenBank (Table 1). This GenBank sample constituted a single representative trombidiform mite species from all those with fully sequenced mitochondria. In geneious prime, we repositioned all GenBank‐sourced mitogenomes with respect to *cox*1. Gene annotations were exported from geneious prime for downstream analysis. The R function gene_map_plot from the genomalicious v0.4 package (Thia & Riginos, 2019) was used to illustrate mitogenomic structure (Appendix: Figures S1–S7).

It is important to note that the reported mitogenome of the chigger mite, *Leptotrombidium pallidum* (GenBank accession: AB180098), has a pseudo‐*rrnS* gene and a duplicated *rrnL* gene (Shao et al., 2005), at respective positions 11772–11984 and 11985–12991, when this mitogenome is repositioned relative to *cox*1 (Appendix: Figure S5d). For phylogenetic analyses, we considered only the nucleotide sequences and gene arrangements of the primary *rrnS* (positions 5401–6001) and *rrnL* (positions 4393–5400), which are homologous to these genes reported for other *Leptotrombidium* species that are repositioned on *cox*1 (Appendix: Figure S5b,c).

In R, we used gene annotations to extract nucleotide sequences from each of the GenBank‐sourced mitogenomes. Nucleotide sequences were reverse complemented when they occurred on the negative strand using biostrings v2.56.0 (Pagès et al., 2017). Each gene was aligned independently using the ClustalW algorithm implemented in the msa v1.20.1 package (Bodenhofer et al., 2015). The best evolutionary model for each gene alignment was established using the modeltest function from the phangorn v2.6.2 package (Schliep, 2011). We considered the “JC,” “F81,” “K80,” “HKY,” and “GTR” models with the possibility of invariable sites (+I) and gamma‐distributed rates (+G). For all protein‐coding and rRNA genes, the best model was GTR + G + I based on AIC scores, and for genes where it was not the best model, it could not be differentiated from the best model based on ΔAIC <10 criteria (Burnham & Anderson, 2004). Therefore, we proceeded with the GTR + G + I model for all genes when constructing nucleotide sequence phylogenies.

Mitogenomic structure, characterized by gene arrangements, was also used to infer phylogenetic relationships. We used 15 genes (protein‐coding and rRNA genes), and we considered their relative order and strand positions as character states. We encoded gene order position as a matrix of dummy variables. For example, let **X_gene_** be a matrix for a focal gene, where the rows are species. **X_gene_** has 30 columns, corresponding to all possible unique combinations of *n* ^th^ ordered positions (1–15) on the *p* ^th^ strand (positive or negative). When the focal gene occurred at position *n* on strand *p*, it was scored as “1” in the respective column of **X_gene_**, and all other columns were scored as “0.” Hence, **X_gene_** encodes binary data for the presence or absence of a focal gene at a specific ordered position and strand on the mitogenome for all species. We concatenated (column‐wise) these binary matrices for each gene to form the matrix, **Y**, which summarized mitogenomic haplotype structure across all species at their protein‐coding and rRNA genes. As defined, the characterization of mitogenomic structural haplotypes in matrix **Y** captures both insertion mutations (that change the relative positions of genes) and inversion mutations (that change the strand on which genes occur and their orientation). Note that because all species were repositioned with respect to *cox*1, and because every species’ *cox*1 gene was on the positive strand, all species were effectively monomorphic for this gene.

Phylogenetic reconstruction was primarily performed using the program mrbayes v3.2.7 (Huelsenbeck & Ronquist, 2001; Ronquist et al., 2012). Prior to analysis with mrbayes, we removed all monomorphic sites (not including gaps and missing sites in the nucleotide sequence alignments). We were left with 12,180 nucleotide character states for nucleotide sequences and 81 character states for the gene arrangements. We fit three models: (1) nucleotide sequences alone, (2) gene arrangements alone, and (3) nucleotide sequences + gene arrangements. For the nucleotide sequence model, all alignments were jointly analyzed, but as discrete partitions. We allowed each gene to have its own set of parameters using the mrbayes command call, “unlink statefreq=(all) revmat=(all) shape=(all) pinvar=(all),” and different evolutionary rates among genes were modeled using the command call, “prset applyto=(all) ratepr=variable.” The GTR + G + I evolutionary model was implemented using the command call, “lset nst=6 rates=invgamma.” For the gene arrangement model, we modeled a gamma distribution of evolutionary rates using the command, “lset rates=gamma.” For the combined model, we used the same parameters described for each respective dataset in their individual models.

For all three Bayesian models, we executed 500,000 generations with the first 25% of generations as burnins, a sample frequency of 500 generations, and diagnostic calculation every 5,000 generations, using 2 independent runs. By generation 500,000, the lagged log‐likelihood difference between simulations had plateaued and fluctuated around 0 (Appendix: Figure S8). By the final generation, the standard deviation of the split frequencies was 0.003, 0.009, and 0.0008 for the nucleotide sequence only, gene arrangement only, and nucleotide sequence + gene arrangement models.

A maximum‐likelihood (ML) tree from nucleotide sequences was also constructed for comparison to Bayesian trees. Nucleotide alignments were concatenated and analyzed with mega x (Kumar et al., 2018). We used a general time reversible model with gamma‐distributed rates (4 discrete gamma categories) and invariant sites. We used complete deletion of gapped and missing sites, setting the ML heuristic to nearest neighbor interchange, with the initial ML tree based on a neighbor‐joining tree. Node support was assessed with 500 bootstrap replicates.

Phylogenetic trees were exported from mrbayes and mega x. Aesthetic embellishments were made using figtree (Rambaut, 2007) and inkscape (Inkscape Project, 2020).

## 3 RESULTS AND DISCUSSION

The mitogenome of *H. destructor*, a major pest of Australian agriculture, has now been fully sequenced and provides a useful resource for comparative genomic and population genetic studies. In Australian agro‐ecosystems, *H. destructor* co‐occurs with several closely related cofamilial species that are morphologically similar, and there is substantial interest on the impact of arthropod predators on these mites (Umina et al., 2004; Weeks & Hoffmann, 2000). Hence, the *H. destructor* mitogenome will be useful for developing metabarcoding tools for the rapid, high‐throughput assessment of mite species diversity and in identifying arthropod predators of *H. destructor* (Cuthbertson et al., 2003; Martin et al., 2015), in Australia.

**FIGURE 1:**
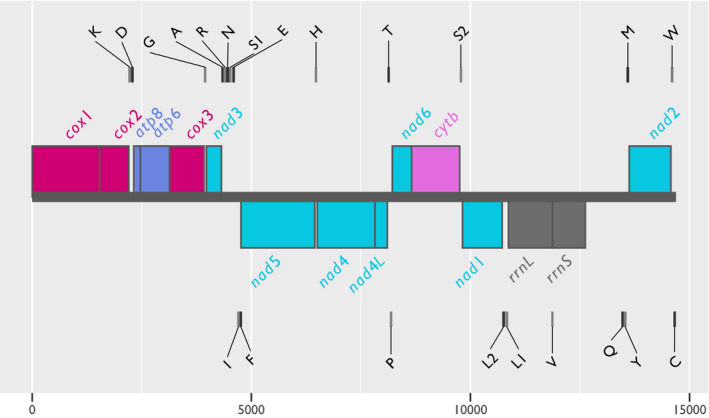
Genetic map of the mitochondrion of *Halotydeus destructor*. The thick, horizontal gray line in the center of the plot illustrates the length of the mitogenome (base position on the x‐axis). Large bars situated on the mitogenome length indicate the position of protein‐coding genes and rRNA genes. Colors of protein‐coding and rRNA genes are as follows: red, *cox* genes; blue, *atp* genes; turquoise, *nad* genes; pink, *cytb*; and gray, rRNA genes. For protein‐coding and rRNA genes, those sitting above the gray line are on the positive strand, and those below the line are on the negative strand. Small gray bars with annotated labels demarcate the positions of tRNA genes

**FIGURE 2:**
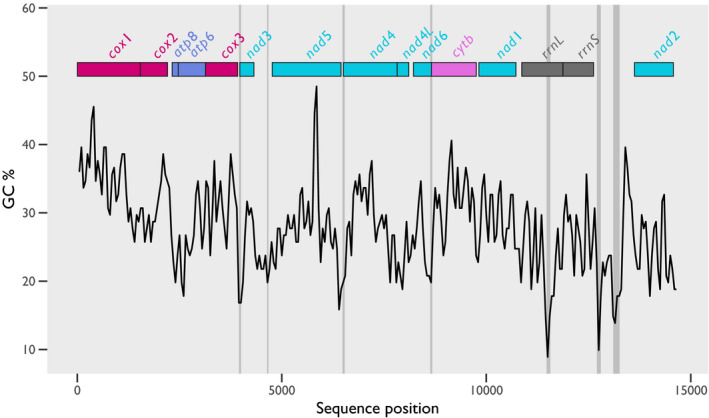
GC content of the *Halotydeus destructor* mitogenome and predicted locations of the replication origin. The x‐axis is the base position, whereas the y‐axis is the GC content (estimated through an overlapping sliding window of 100 bp). The black line tracks the GC content across the mitogenome. The vertical gray bars indicate the predicted locations of replication origins. The horizontal bars at the top of the plot illustrate the positions of protein‐coding and rRNA genes. Colors of protein‐coding and rRNA genes are as follows: red, *cox* genes; blue, *atp* genes; turquoise, *nad* genes; pink, *cytb*; and gray, rRNA genes

The mitogenome of *H. destructor* is 14,691 bp long and has a GC content of 27.87%. It has 13 protein‐coding genes, 2 rRNA genes, and 22 tRNAs (includes two putative tRNA genes for Leu and Ser) (Figure 1). A total of 8 replication origins were predicted by mitos2 (Figure 2). Based on the joint expectation that mitochondrial control regions occur in noncoding and low GC regions, the most likely candidate for the *H. destructor* control region would be between *rrnS* and *nad*2 (position 12,623–13,624, a width of 1,001 bp). This region contains two predicted replication origins (Figure 2). Our mitogenome of *H. destructor* has been submitted to GenBank under the Accession, MZ357702. Genetic features of the *H. destructor* mitogenome are tabulated in Table S1.

**FIGURE 3:**
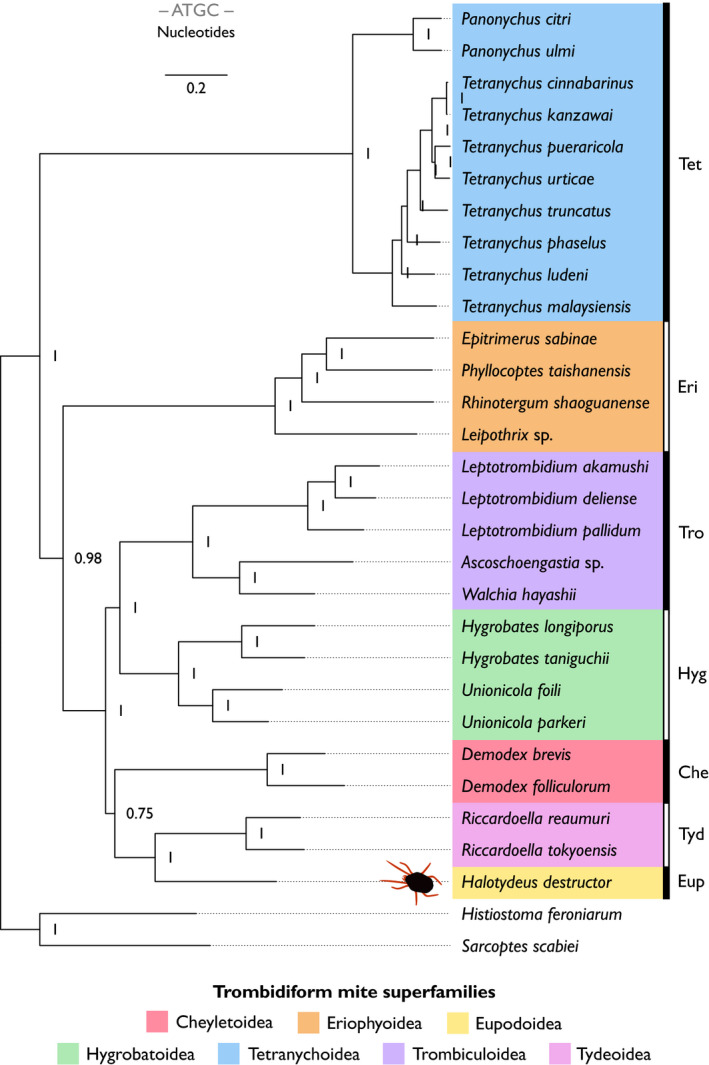
Bayesian molecular phylogeny (nucleotide sequences) of mitochondrial protein‐coding and rRNA genes. Superfamilies of trombidiform mites are highlighted with different colors (see legend). To the right of species names, abbreviations denote trombidiform superfamilies: Che, Cheyletoidea; Eri, Eriophyoidea; Eup, Eupodoidea; Hyg, Hygrobatoidea; Tet, Tetranychoidea; Tro, Trombiculoidea; Tyd, Tydeoidea. The sarcoptiform mites, *Histiostoma feroniarum* and *Sarcoptes scabiei*, were used as out‐group taxa. Node labels denote posterior probability support. The scale bar (top of plot) is the number of substitutions per unit length

**FIGURE 4:**
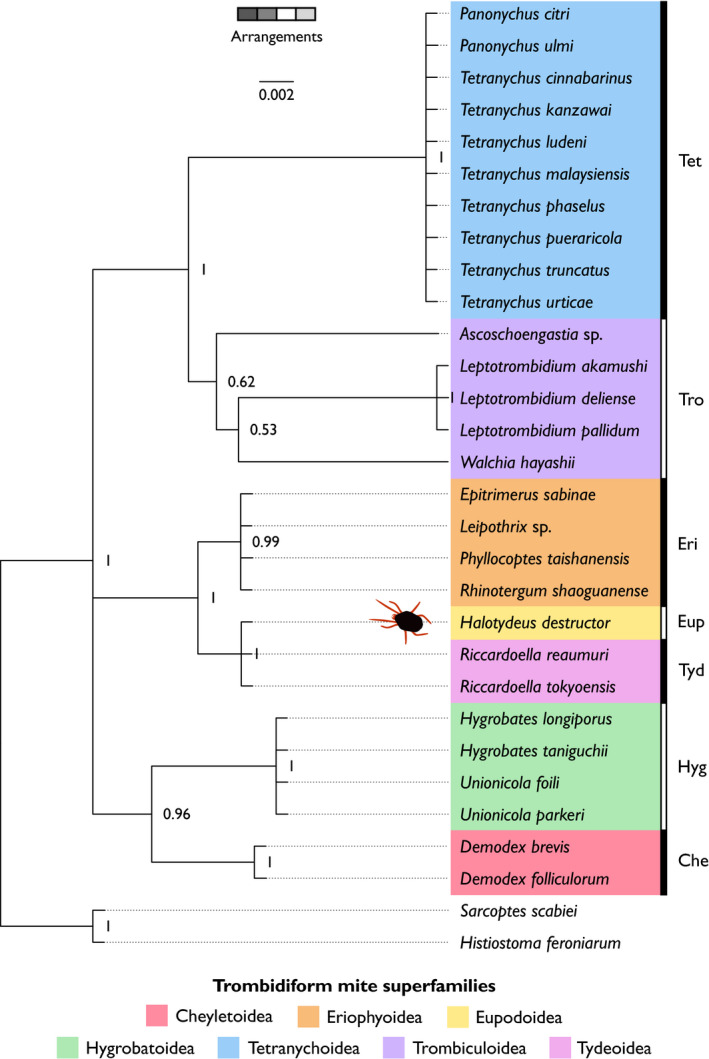
Bayesian trait phylogeny (gene arrangements) of mitochondrial protein‐coding and rRNA genes. Superfamilies of trombidiform mites are highlighted with different colors (see legend). To the right of species names, abbreviations denote trombidiform superfamilies: Che, Cheyletoidea; Eri, Eriophyoidea; Eup, Eupodoidea; Hyg, Hygrobatoidea; Tet, Tetranychoidea; Tro, Trombiculoidea; Tyd, Tydeoidea. The sarcoptiform mites, *Histiostoma feroniarum* and *Sarcoptes scabiei*, were used as out‐group taxa. Node labels denote posterior probability support. The scale bar (top of plot) is the number of substitutions per unit length

**FIGURE 5:**
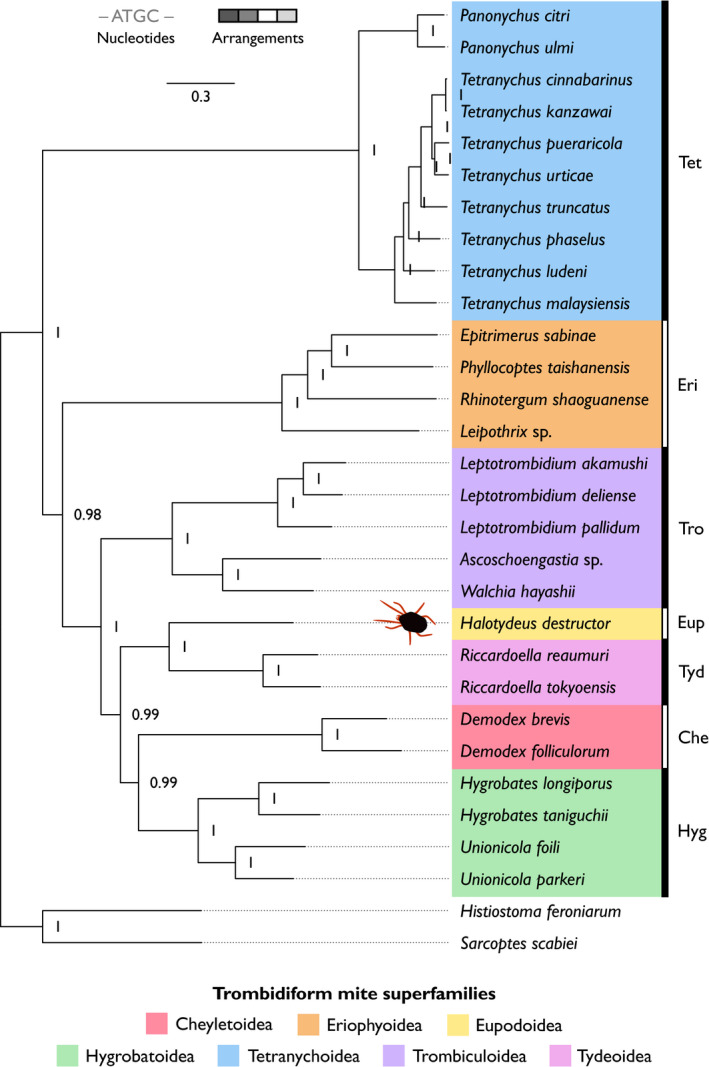
Bayesian combined molecular and trait phylogeny (nucleotide sequences + gene arrangements) of mitochondrial protein‐coding and rRNA genes. Superfamilies of trombidiform mites are highlighted with different colors (see legend). To the right of species names, abbreviations denote trombidiform superfamilies: Che, Cheyletoidea; Eri, Eriophyoidea; Eup, Eupodoidea; Hyg, Hygrobatoidea; Tet, Tetranychoidea; Tro, Trombiculoidea; Tyd, Tydeoidea. The sarcoptiform mites, *Histiostoma feroniarum* and *Sarcoptes scabiei*, were used as out‐group taxa. Node labels denote posterior probability support. The scale bar (top of plot) is the number of substitutions per unit length

Using nucleotide sequences and gene arrangement information from protein‐coding and rRNA genes, we examined the evolutionary relationships between *H. destructor* and other Trombidiformes with complete mitogenomes (for genetic maps, see Appendix: Figures S1–S7). Gene arrangements can provide alternative characters to track evolutionary relationships among different taxa (Froufe et al., 2020; Gong et al., 2019; Inoue et al., 2003; Kutyumov et al., 2021). Prior analyses of mitogenomic structural haplotypes in trombidiform mites have focused on a comparatively smaller number of species to that analyzed in our study (Edwards et al., 2011; Li & Xue, 2019; Palopoli et al., 2014; Shao et al., 2005; Xue et al., 2017; Xue et al., 2016). Despite generalities between phylogenies constructed from nucleotide sequences (Figure 3), gene arrangements (Figure 4), or both (Figure 5), there were some major differences between them with respect to the placement of certain superfamilies of the Trombidiformes. Additionally, whereas the nucleotide sequence and combined phylogeny both exhibited topologies of nested bifurcating trees among the representative trombidiform superfamilies (Figures 3, 5), the gene arrangement phylogeny split the superfamilies into three clades that came together at a trifurcating node (Figure 4). Hence, gene arrangements, as defined in this study, were alone not able to completely resolve relationships among Trombidiformes. A maximum‐likelihood tree of nucleotide sequences (Figure S9) produced similar results to its Bayesian counterpart (Figure 3), so we focus our discussion on results from the Bayesian analyses, linking our results to prior research and hypotheses on the evolutionary relationships among superfamilies in the Trombidiformes.

**FIGURE 6:**
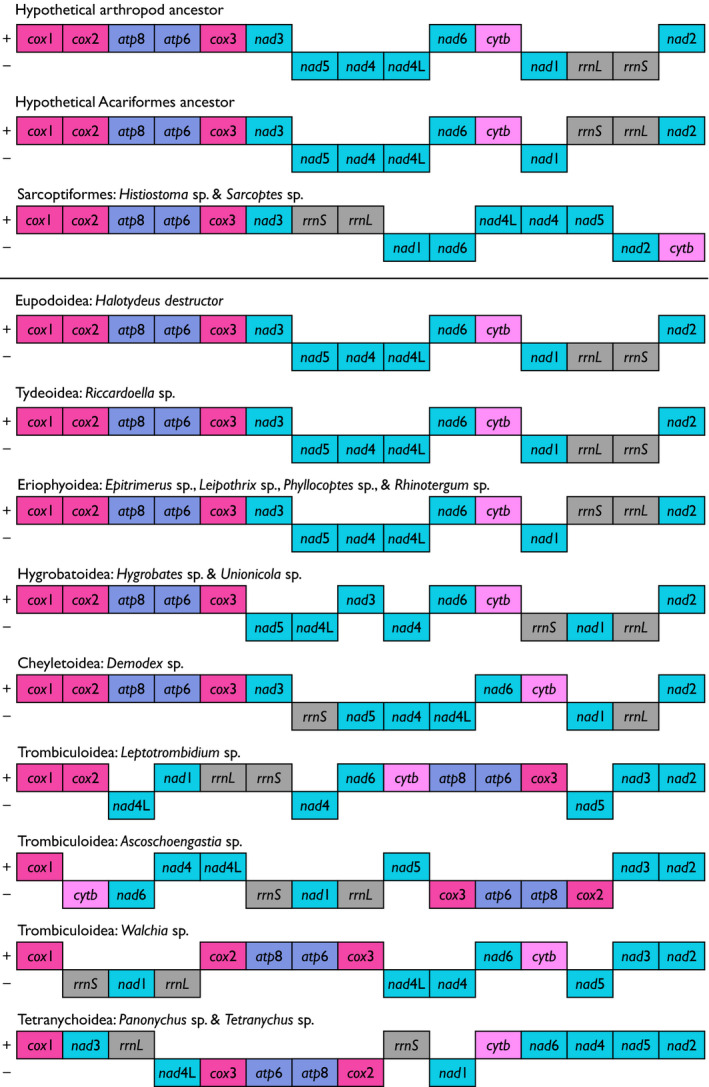
Mitochondrial gene arrangements for protein‐coding and rRNA genes among Trombidiformes, Sarcoptiformes, the hypothetical Acariformes ancestor, and the hypothetical arthropod ancestor. Each box represents a gene. Colors of protein‐coding and rRNA genes are as follows: red, *cox* genes; blue, *atp* genes; turquoise, *nad* genes; pink, *cytb*; and gray, rRNA genes. The “+” and the “–” symbols denote the positive and negative strands, respectively. The black horizontal line separates trombidiform mite haplotypes (below line) from sarcoptiform mite haplotypes and the hypothesized ancestral acariforme and arthropod haplotypes (above line). The trombidiform mitochondrial gene arrangements are ordered (top to bottom) with respect to their structural similarity to *Halotydeus destructor* (superfamily Eupodoidea). The ancestral acariform and arthropod haplotypes are taken from Xue et al. (2016)

*Halotydeus destructor* belongs to the superfamily Eupodoidea. Our phylogenies derived from nucleotide sequences (Figure 3), gene arrangements (Figure 4), or both (Figure 5), always placed *H. destructor* as the sister taxon to *Riccardoella* species, which are from the superfamily Tydeoidea. This relationship was always strongly supported (≥99%). *Halotydeus destructor* and the *Riccardoella* species exhibited complete synteny in their gene arrangements; that is, they possessed the exact same haplotype in the structural organization of their protein‐coding and rRNA genes (Figure 6). Our observations run counter to prior hypotheses that Tydeoidea is sister to Eriophyoidea and that Eupodoidea is paraphyletic to the Eriophyoidea–Tydeoidea sister pair, based on morphological data (Lindquist, 1998; Mironov & Bochkov, 2009; Qin, 1996). Other more recent molecular studies have noted the Eupodoidea–Tydeoidea sister pairing (Klimov et al., 2018). We also found that the Eupodoidea−Tydeoidea mitogenomic haplotype is identical to the hypothesized ancestral arthropod haplotype (Shao et al., 2005; Xue et al., 2016), indicating a very high level of conservatism in the gene arrangements of the Eupdoidea and Tydeoidea relative to other trombidiform superfamilies (Figure 6). Indeed, this conservatism in mitogenomic structure has persisted over more than 400 million years, the estimated emergence of the Acariformes, the superorder containing the Trombidiformes (Dabert et al., 2010; Jeyaprakash & Hoy, 2009; Xue et al., 2017).

With respect to the superfamily Eriophyoidea, contemporaneous molecular studies have observed two contrasting patterns: (i) Eriophyoidea basal to all Trombidiformes (18S + 28S rRNA and *cox*1) and (ii) Eriophyoidea as a closely related taxon to the infraorder Eupodina (*ef1‐α*, *srp*54, *hsp*70), which includes Eupodoidea and Tydeoidea (Klimov et al., 2018). We observed two contrasting relationships in our data. Placement of Eriophyoidea as the out‐group to the clade containing species representing the superfamilies Cheyletoidea, Eupodoidea, Hygrobatoidea, Trombiculoidea, and Tydeoidea was strongly supported by both the nucleotide phylogeny (98%; Figure 3) and the combined phylogeny (95%; Figure 5). Such a placement is consistent with a hypothesis of a more basal origin of the superfamily Eriophyoidea, but still evolving within the Trombidiformes. Yet we also observed near‐identical gene arrangements between Eriophyoidea with Eupodoidea and Tydeoidea (Figure 6). The only difference between the Eriophyoidea structural haplotype and that observed in Eupodoidea and Tydeoidea was the order of the *rrnS* and *rrnL* genes and the strands where these genes occurred (Figure 6). Consequently, there was very strong support (100%) for Eriophyoidea as the sister taxon to Eupodoidea–Tydeoidea pair in our phylogeny of gene arrangements (Figure 4), which is consistent with a hypothesis of a close affinity between the superfamily Eriophyoidea and the infraorder Eupodina (Lindquist, 1998; Qin, 1996). Hence, gene arrangements alone painted a very different picture of the origin of the Eriophyoidea relative to their underlying nucleotide sequences.

In this study, our representative species of the superfamily Cheyletoidea were those of the genus *Demodex*. The Cheyletoidea belong to the infraorder Eleutherengona, which includes the superfamily Tetranychoidea, and the Eleutherengona mites are expected to be paraphyletic to those in the infraorder Eupodina (Klimov et al., 2018; Lindquist, 1998; Mironov & Bochkov, 2009; Qin, 1996). Previous molecular studies have reported contrasting placements of Cheyletoidea species, both within clades containing Hygrobatoidea and Eupodoidea (Dabert et al., 2016), and in clades paraphyletic to the Hygrobatoidea and Eupodoidea (Klimov et al., 2018). We observed two distinct placements of *Demodex* species. The nucleotide phylogeny exhibited poor support (75%) for *Demodex* species positioned paraphyletic to the Eupodoidea–Tydeoidea pair (Figure 3). Contrastingly, there was very strong support for *Demodex* species as the sister taxon to the Hygrobatoidea mites in phylogenies inferred from gene arrangements (96%; Figure 4) and the combined phylogeny (98%; Figure 5). Clearly, mites of the *Demodex* genus have unusual mitogenomes relative to members of their superfamily, Cheyletoidea, and exhibit greater similarity to Eupodoidea, Tydeoidea, and Hygrobatoidea mitogenomes than they do to Tetranychoidea. Unfortunately, mitogenomes of other Cheyletoidea species are not available, so we were unable to draw comparisons with other members of this superfamily.

Across mite superfamilies sampled with multiple representative genera, only the Trombiculoidea exhibited heterogeneity among genera in their gene arrangements. Each of the Trombiculoidea genera (*Leptotrombidium*, *Ascoschoengastia*, and *Walchia*) possessed a distinct structural haplotype for their protein‐coding and rRNA genes (Figure 6). Moreover, within the genus *Leptotrombidium*, two unique haplotypes exist: one with an additional pseudo‐*rrnS* gene and duplicated *rrnL* gene that is found in *L. pallidum* (Appendix: Figure S5d) and the other with single copies of the rRNA genes that is found in other *Leptotrombidium* species (Appendix: Figure S5b,c) (Shao et al., 2005, 2006). The inferred evolutionary relationships of Trombiculoidea to other superfamilies in the Trombidiformes varied across all three phylogenies. In the nucleotide phylogeny, we observed strong support (100%) for a Trombiculoidea–Hygrobatoidea pairing that was paraphyletic to a clade containing Cheyletoidea, Eupodoidea, and Tydeoidea (Figure 3). However, the combined data strongly supported (100%) Trombiculoidea placed paraphyletic to a clade containing Cheyletoidea, Hygrobatoidea, Eupodoidea, and Tydeoidea. Furthermore, when exclusively considering gene arrangements, the Trombiculoidea were placed as the sister taxon to the Tetranychoidea (Figure 4). This strongly supported (100%) Tetranychoidea–Trombiculoidea pairing in the gene arrangement phylogeny potentially manifested because both these superfamilies have quite divergent structural haplotypes relative to other representatives of the trombidiform superfamilies sampled in this study (Figure 6).

Finally, we note the polyphyly between *Tetranychus cinnabarinus*, the carmine spider mite, and *Tetranychus urticae*, the two‐spotted spider mite, in phylogenies containing nucleotide sequences (Figures 3, 5). *Tetranychus cinnabarinus* is now considered synonymous with *T. urticae*, and prior erroneous taxonomic separation was attributable to high levels of polymorphism in *T. urticae* (Auger et al., 2013), although the two forms can show some transcriptional and genomic differences (Huo et al., 2021). In principle, *T. cinnabarinus* and *T. urticae* should be a sister pair; however, the fact that we did not observe such a sister pairing in our analyses suggests taxonomic mislabeling may have occurred for the accession of *T. cinnabarinus*, which we found to be most closely related to *Tetranychus kanzawai*, the kanzawa spider mite.

In conclusion, we present the complete mitochondrial sequence of *H. destructor*, which will aid future population genetic investigations in this major pest of Australian agriculture, and arthropod diversity monitoring in Australian agro‐ecosystems. We show that gene arrangements in protein‐coding and rRNA genes provide complementary information—to that obtained from nucleotide sequences alone—for inferring the evolutionary relationships among superfamilies in the Trombidiformes. This study provides the most comprehensive assessment of mitogenomic structure in the Trombidiformes to date. However, because we focused on available complete mitogenomic sequences, we provide only shallow sampling of the taxonomic breath within and among superfamilies, except for the Tetranychoidea. Future works including many more superfamilies will impart further insights into how gene arrangements can help resolve taxonomic uncertainty among trombidiform mites.

## 4 R PACKAGES

ape v5.4‐1 (Paradis et al., 2004).

data.table v1.13.0 (Dowle & Srinivasan, 2019).

doparallel v1.0.16 (Microsoft Corporatin & Steve Weston, 2019).

bio3d v2.4‐1 (Grant et al., 2006).

biostrings v2.56.0 (Pagès et al., 2017).

genomalicious v0.4 (Thia & Riginos, 2019).

ggpubr v0.4.0 (Kassambara, 2020).

phangorn v2.6.2 (Schliep, 2011).

msa v1.20.1 (Bodenhofer et al., 2015).

readxl v1.3.1 (Wickham & Bryan, 2019).

tidyverse v1.3.0 (Wickham et al., 2019).

## CONFLICT OF INTEREST

The authors have no conflicts of interest associated with this study.

## AUTHOR CONTRIBUTION

**Joshua Thia:** Conceptualization (lead); Data curation (lead); Formal analysis (lead); Investigation (lead); Methodology (lead); Project administration (lead); Validation (lead); Visualization (lead); Writing‐original draft (lead); Writing‐review & editing (lead). **Neil D. Young:** Formal analysis (supporting); Methodology (supporting); Writing‐review & editing (supporting). **Pasi K. Korhnen:** Formal analysis (supporting); Methodology (supporting); Writing‐review & editing (supporting). **Qiong Yang:** Data curation (supporting); Methodology (supporting); Project administration (supporting); Resources (supporting); Writing‐review & editing (supporting). **Robin B. Gasser:** Formal analysis (supporting); Methodology (supporting); Writing‐review & editing (supporting). **Paul A. Umina:** Conceptualization (supporting); Funding acquisition (equal); Investigation (supporting); Project administration (supporting); Resources (equal); Writing‐review & editing (supporting). **Ary A. Hoffmann:** Conceptualization (supporting); Funding acquisition (equal); Investigation (supporting); Methodology (supporting); Project administration (supporting); Resources (equal); Writing‐review & editing (supporting).

## Supporting information

Figures S1‐S9 and Table S1

## Data Availability

The mitogenomic sequence of *Halotydeus destructor* is available through GenBank: Accession MZ357702. Scripts and associated data for analyses presented in this study have been archived in Dryad: doi.org/10.5061/dryad.37pvmcvkj (Thia, 2021).
