# Supplementary material for: The mitogenome of Halotydeus destructor (Tucker) and its relationships with other trombidiform mites as inferred from nucleotide sequences and gene arrangements
Source: Ecol Evol. 2021 Sep 22;11(20):14162–74. doi: 10.1002/ece3.8133 (PMC8525180; doi:10.1002/ece3.8133)
Supplement: Supplementary file 1 — Figures S1‐S9 and Table S1 [file ECE3-11-14162-s001.pdf]

SUPPLEMENTARY INFORMATION

The mitogenome of *Halotydeus destructor* (Tucker) and its relationships with other trombidiform mites as inferred from nucleotide sequences and gene arrangements

Joshua A. Thia [1§], Neil D. Young [2], Pasi K. Korhnen [2], Qiong Yang [1], Robin B. Gasser [2], Paul A. Umina [1,3], Ary A. Hoffmann [1]

[1] Bio 21 Institute, School of BioSciences, The University of Melbourne, VIC, Australia

[2] Department of Veterinary Biosciences, Melbourne Veterinary School, Faculty of Veterinary and Agricultural Sciences, The University of Melbourne, VIC, Australia

[3] Cesar Australia, Brunswick, VIC, Australia

[§] Corresponding author: josh.thia@live.com, joshua.thia@unimelb.edu.au

APPENDIX FIGURES

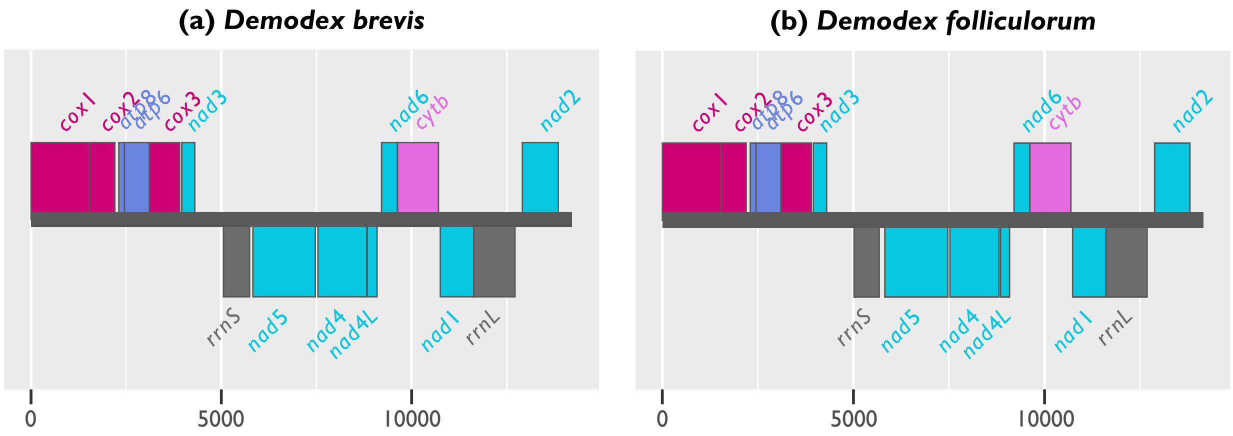

Figure S1. Genetic maps for the mitochondria of the Cheyletoidea superfamily species (Trombidiformes). The thick, horizontal grey line in the center of the plot illustrates the length of the mitogenome (base position on the x-axis). Large bars situated on the mitogenome length indicate the position of protein-coding genes and rRNA genes, with colours indicating gene type: red = *cox* genes; blue = *atp* genes; turquoise = *nad* genes; pink = *cytb*; and grey = rRNA genes. Those genes sitting above the grey line are on the positive strand, and those below the line are on the negative strand

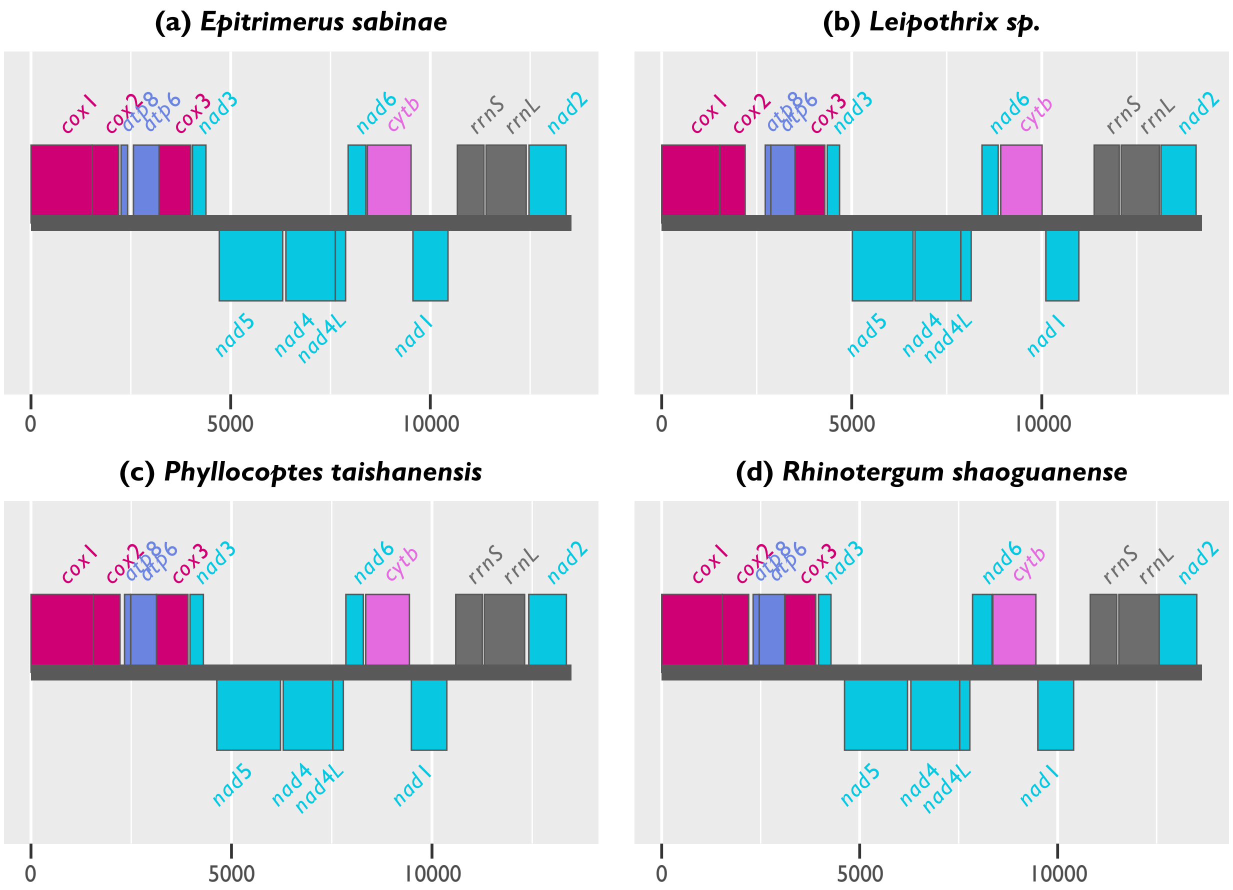

Figure S2. Genetic maps for the mitochondria of the Eriophyoidea superfamily species (Trombidiformes). The thick, horizontal grey line in the center of the plot illustrates the length of the mitogenome (base position on the x-axis). Large bars situated on the mitogenome length indicate the position of protein-coding genes and rRNA genes, with colours indicating gene type: red = *cox* genes; blue = *atp* genes; turquoise = *nad* genes; pink = *cytb*; and grey = rRNA genes. Those genes sitting above the grey line are on the positive strand, and those below the line are on the negative strand.

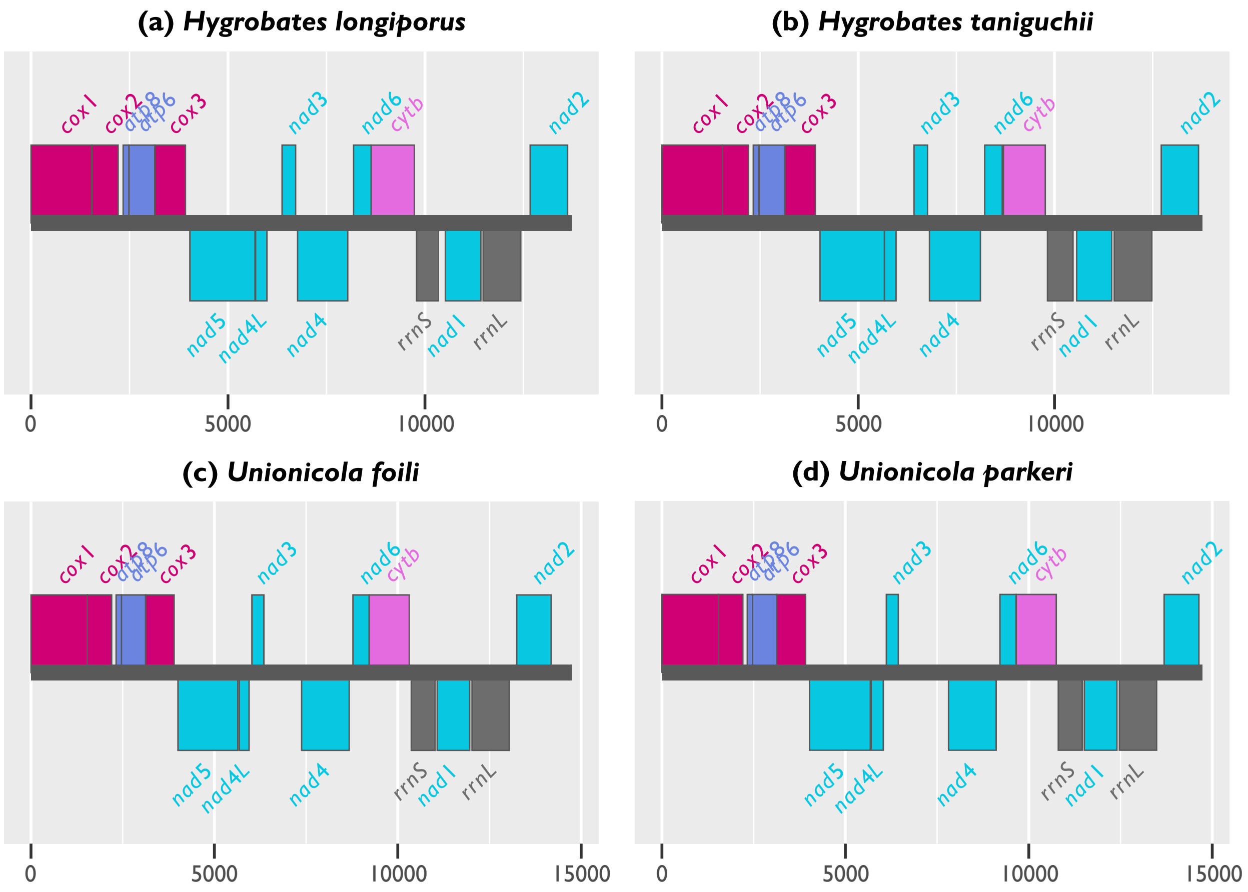

Figure S3. Genetic maps for the mitochondria of the Hygrobatoidea superfamily species (Trombidiformes). The thick, horizontal grey line in the center of the plot illustrates the length of the mitogenome (base position on the x-axis). Large bars situated on the mitogenome length indicate the position of protein-coding genes and rRNA genes, with colours indicating gene type: red = *cox* genes; blue = *atp* genes; turquoise = *nad* genes; pink = *cytb*; and grey = rRNA genes. Those genes sitting above the grey line are on the positive strand, and those below the line are on the negative strand.

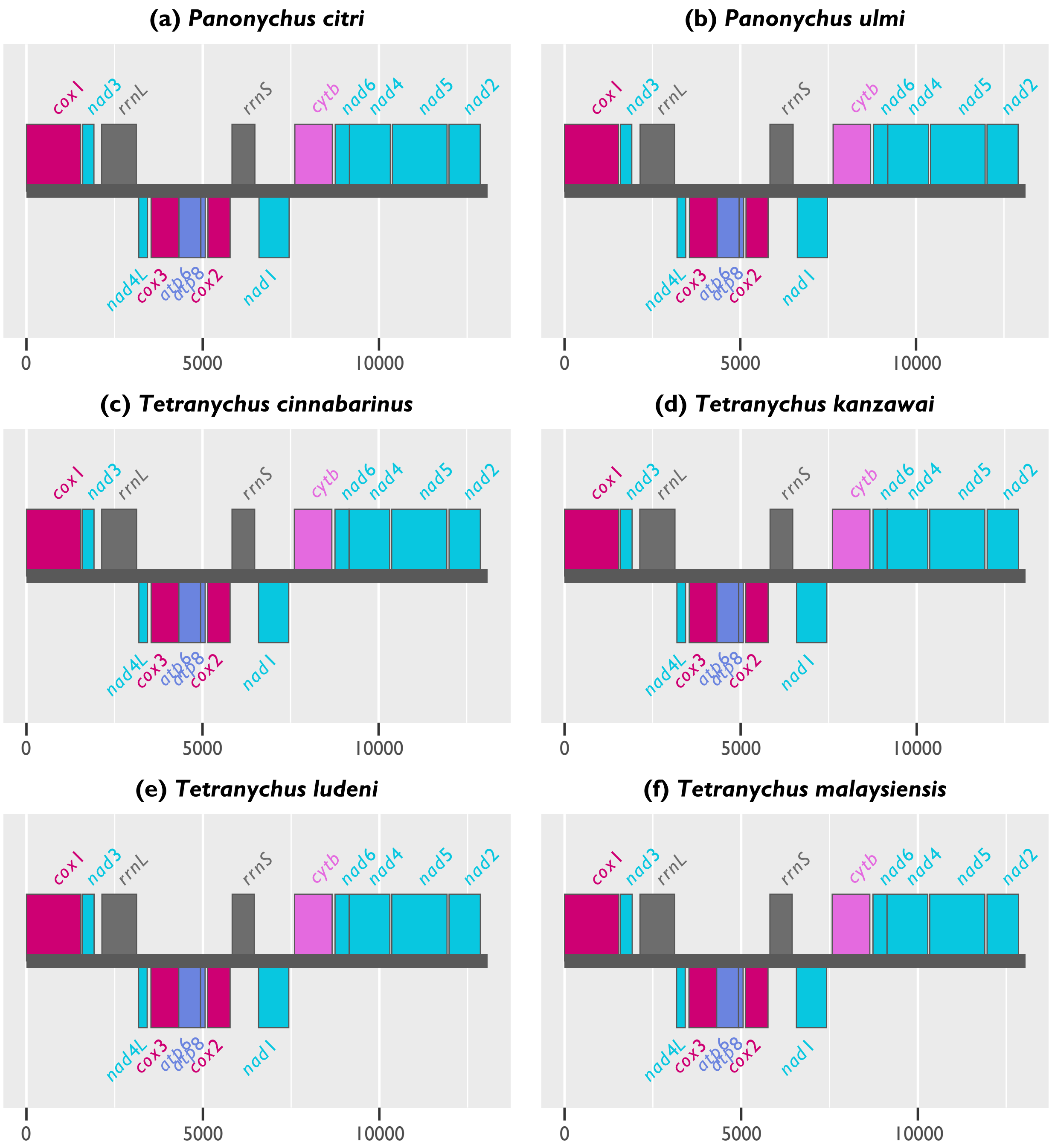

Figure S4.1. Genetic maps for the mitochondria of the Tetranychoidea superfamily species (Trombidiformes). The thick, horizontal grey line in the center of the plot illustrates the length of the mitogenome (base position on the x-axis). Large bars situated on the mitogenome length indicate the position of protein-coding genes and rRNA genes, with colours indicating gene type: red = *cox* genes; blue = *atp* genes; turquoise = *nad* genes; pink = *cytb*; and grey = rRNA genes. Those genes sitting above the grey line are on the positive strand, and those below the line are on the negative strand.

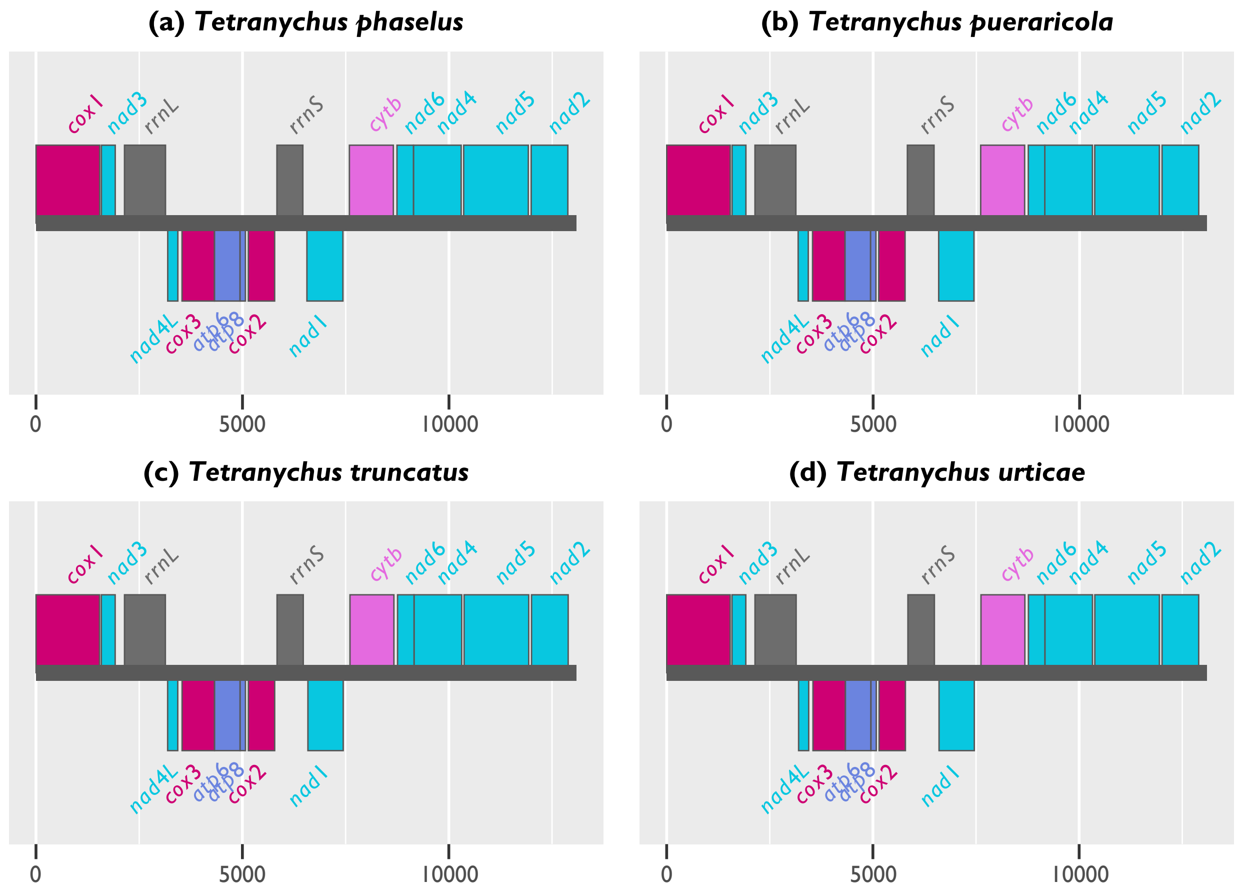

Figure S4.2. Genetic maps for the mitochondria of the Tetranychoidea superfamily species (Trombidiformes). The thick, horizontal grey line in the center of the plot illustrates the length of the mitogenome (base position on the x-axis). Large bars situated on the mitogenome length indicate the position of protein-coding genes and rRNA genes, with colours indicating gene type: red = *cox* genes; blue = *atp* genes; turquoise = *nad* genes; pink = *cytb*; and grey = rRNA genes. Those genes sitting above the grey line are on the positive strand, and those below the line are on the negative strand.

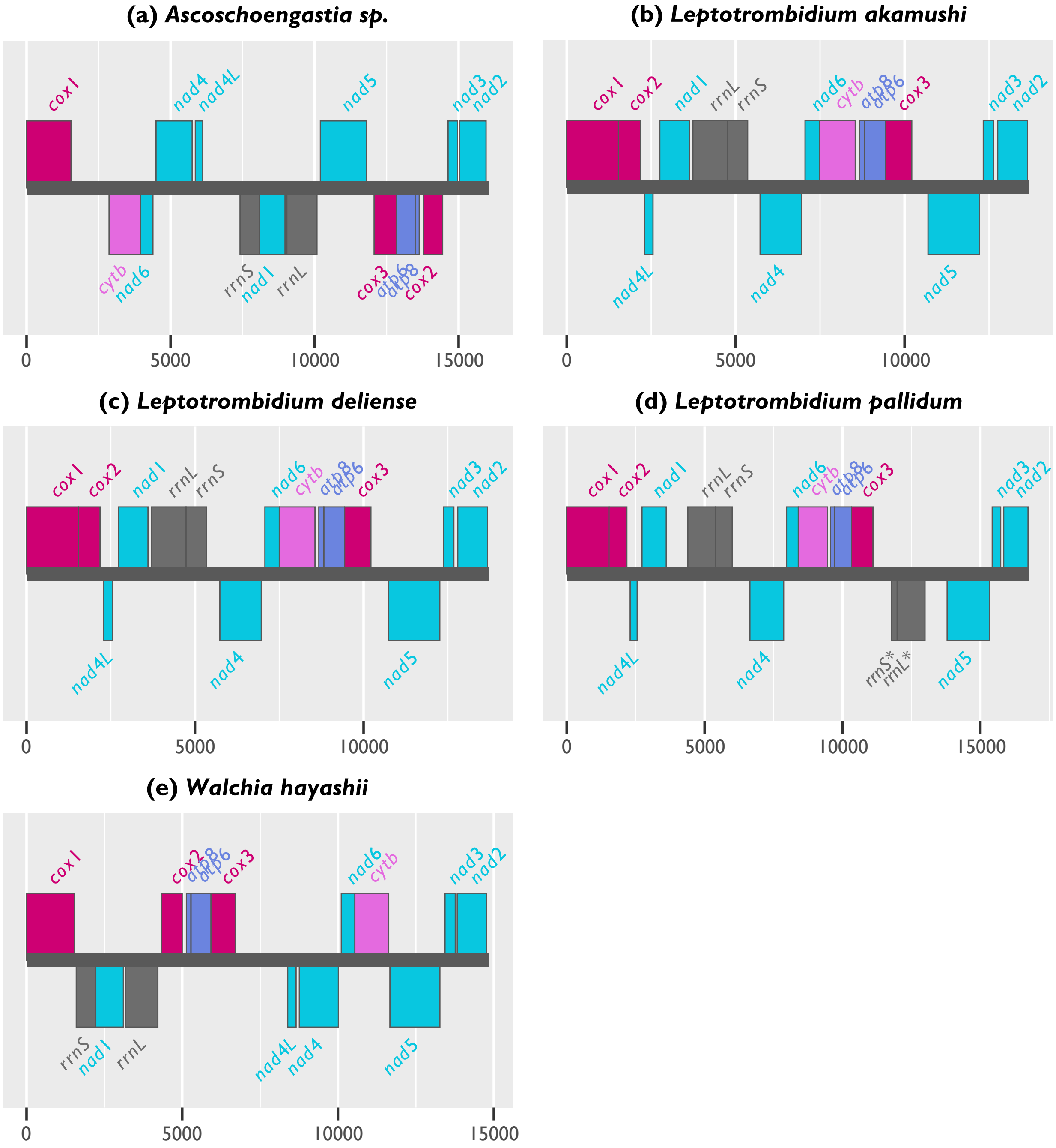

Figure S5. Genetic maps for the mitochondria of the Trombiculoidea superfamily species (Trombidiformes). The thick, horizontal grey line in the center of the plot illustrates the length of the mitogenome (base position on the x-axis). Large bars situated on the mitogenome length indicate the position of protein-coding genes and rRNA genes, with colours indicating gene type: red = cox genes; blue = *atp* genes; turquoise = *nad* genes; pink = *cytb*; and grey = rRNA genes. Those genes sitting above the grey line are on the positive strand, and those below the line are on the negative strand. Note, in (d), *rrnS** is a pseudo *rrnS* gene, and *rrnL** is a duplicated *rrnL* gene.

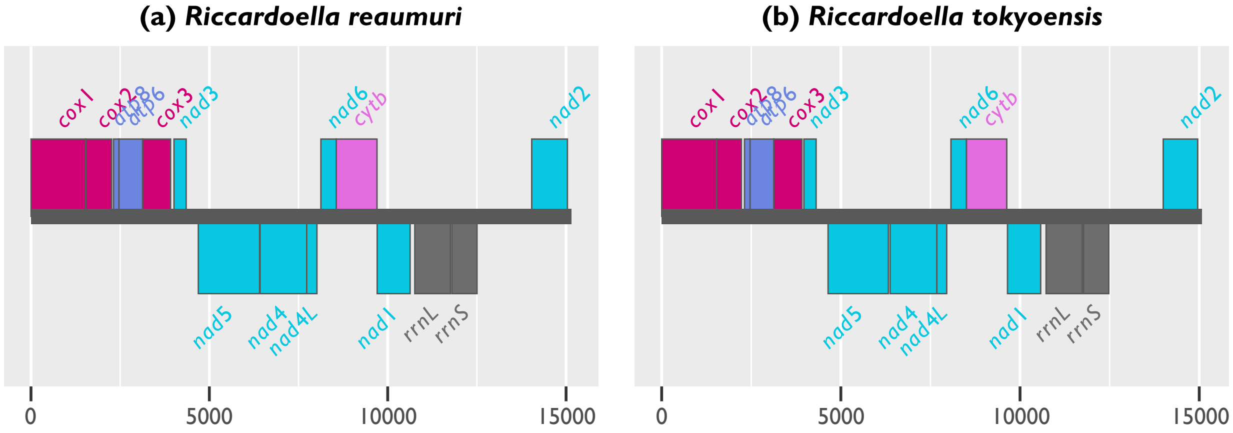

Figure S6. Genetic maps for the mitochondria of the Tydeoidea superfamily species (Trombidiformes). The thick, horizontal grey line in the center of the plot illustrates the length of the mitogenome (base position on the x-axis). Large bars situated on the mitogenome length indicate the position of protein-coding genes and rRNA genes, with colours indicating gene type: red = *cox* genes; blue = *atp* genes; turquoise = *nad* genes; pink = *cytb*; and grey = rRNA genes. Those genes sitting above the grey line are on the positive strand, and those below the line are on the negative strand.

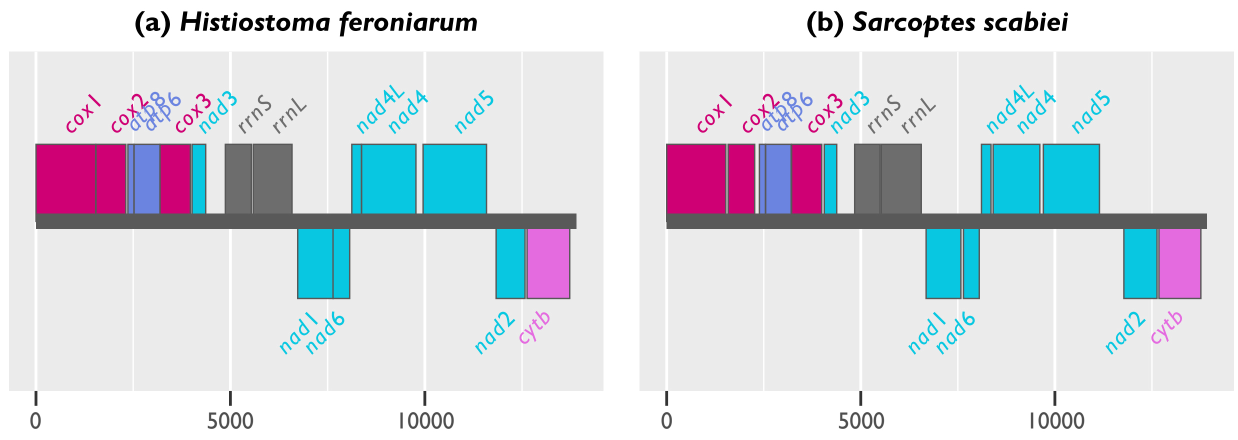

Figure S7. Genetic maps for the mitochondria of species from the Sarcoptiformes. The thick, horizontal grey line in the center of the plot illustrates the length of the mitogenome (base position on the x-axis). Large bars situated on the mitogenome length indicate the position of protein-coding genes and rRNA genes, with colours indicating gene type: red = *cox* genes; blue = *atp* genes; turquoise = *nad* genes; pink = *cytb*; and grey = rRNA genes. Those genes sitting above the grey line are on the positive strand, and those below the line are on the negative strand.

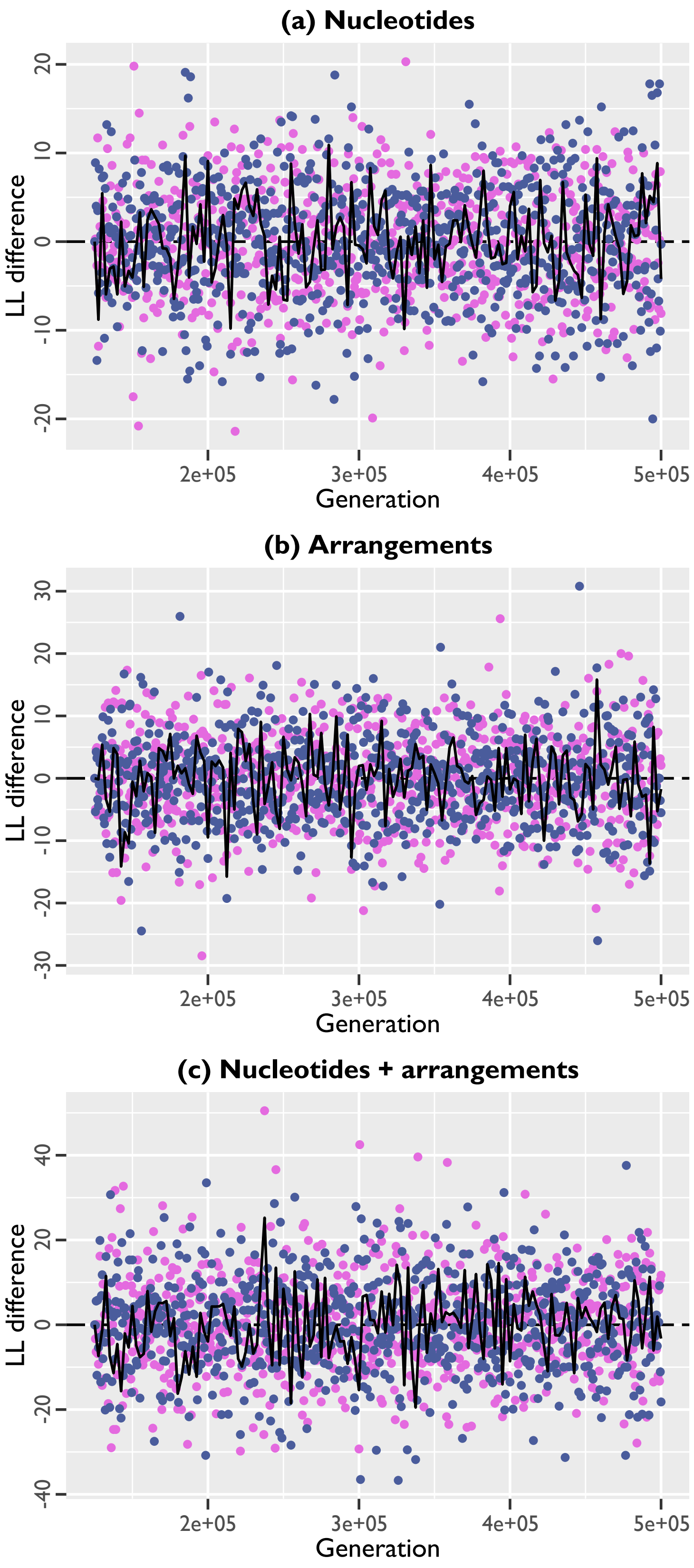

Figure S8. Changes in log-likelihood across Bayesian simulations of phylogenetic relationships (inferred from MrBayes) derived from mitochondrial protein-coding and rRNA genes and their: (a) nucleotide sequences, (b) gene arrangements, or (c) nucleotide sequences + gene arrangements. The x-axis is the simulation generation from the end of the burnin period (125,000) to the final generation (500,000). The y-axis is the lagged log-likelihood (LL) difference between diagnostic calculation points (every 5,000 generations). Points are coloured by run (one of two). The dotted line denotes a difference of zero, that is, the sampled diagnostic point does not have a different log-likelihood to the previous diagnostic point. The solid line tracks the mean of the two runs.

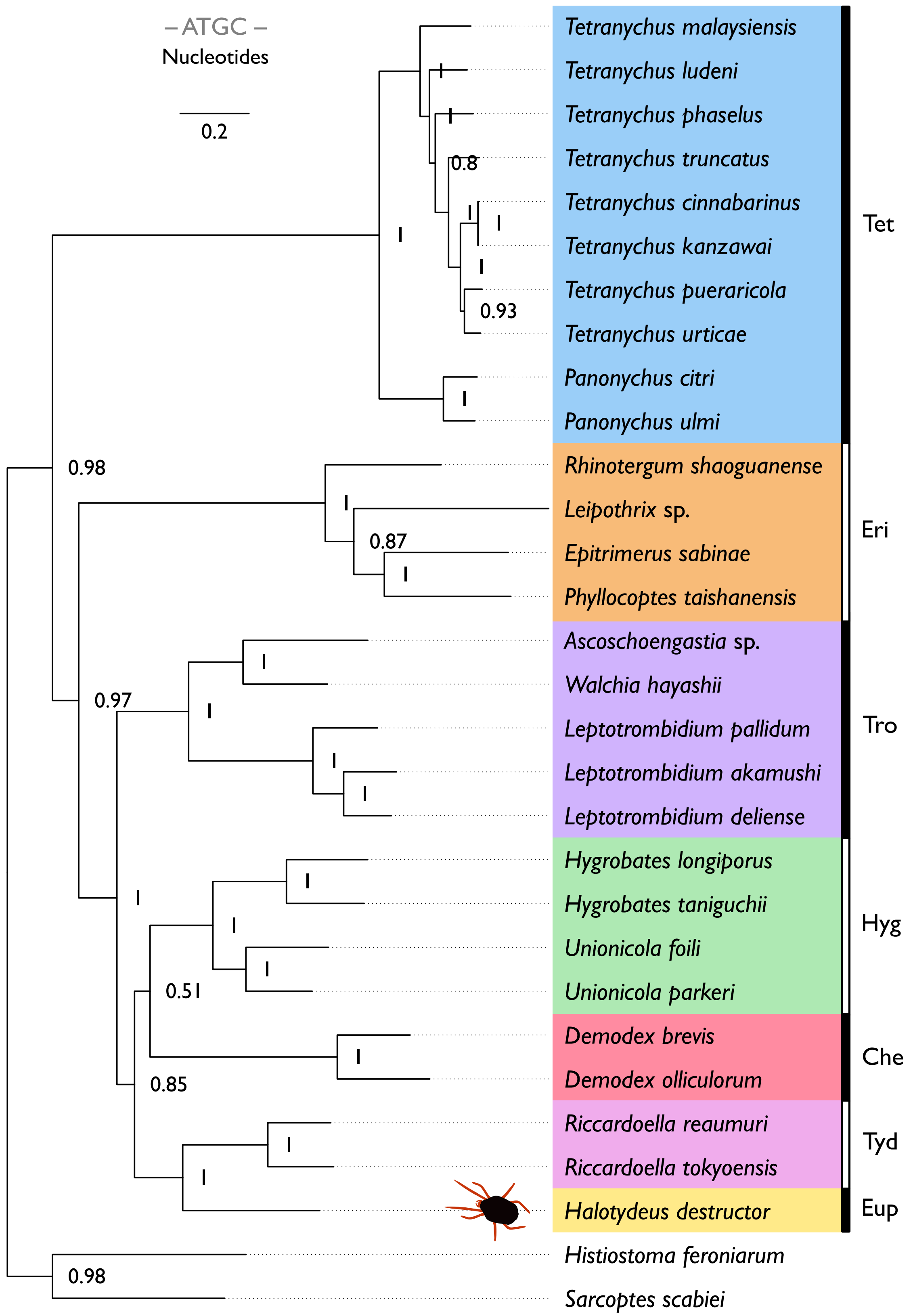

Trombidiform mite superfamilies

Cheyletoidea | Eriophyoidea | Eupodoidea | Hygrobatoidea | Tetranychoidea | Trombiculoidea | Tydeoidea

Figure S9. Maximum likelihood molecular phylogeny (nucleotide sequences) of mitochondrial protein-coding and rRNA genes. Superfamilies of trombidiform mites are highlighted with different colours (see legend). To the right of species names, abbreviations denote trombidiform superfamilies: Che = Cheyletoidea; Eri = Eriophyoidea; Eup = Eupodoidea; Hyg = Hygrobatoidea; Tet = Tetranychoidea; Tro = Trombiculoidea; Tyd = Tydeoidea. The sarcoptiform mites, *Histiostoma feroniarum* and *Sarcoptes scabiei*, were used as outgroup taxa. Node labels denote bootstrap support (n = 500). The scale bar (top of plot) is the number of substitutions per unit length.

APPENDIX TABLES

Table S1. Genetic features of the *Halotydeus destructor* mitogenome.

Genetic feature	Start	End	Length	Strand
*cox*1	1	1539	1539	1
*cox*2	1539	2205	667	1
*atp*8	2318	2473	156	1
*atp*6	2467	3135	669	1
*cox*3	3135	3920	786	1
*nad*3	3974	4318	345	1
*nad*5	4766	6449	1684	−1
*nad*4	6505	7821	1317	−1
*nad*4L	7823	8104	282	−1
*nad*6	8218	8658	441	1
cytb	8658	9755	1098	1
*nad*1	9820	10725	906	−1
*nad*2	13624	14574	951	1
rrnL	10868	11873	1006	−1
rrnS	11876	12623	748	−1
tRNA-K	2194	2258	65	1
tRNA-D	2258	2317	60	1
tRNA-G	3919	3973	55	1
tRNA-A	4317	4366	50	1
tRNA-R	4368	4428	61	1
tRNA-N	4428	4492	65	1
tRNA-S1	4494	4549	56	1
tRNA-E	4566	4627	62	1
tRNA-I	4677	4734	58	−1
tRNA-F	4735	4789	55	−1
tRNA-H	6450	6504	55	1
tRNA-T	8107	8161	55	1
tRNA-P	8161	8216	56	−1
tRNA-S2	9757	9810	54	1
tRNA-L2	10726	10789	64	−1
tRNA-L1	10798	10862	65	−1
tRNA-Q	13444	13498	55	−1
tRNA-Y	13500	13562	63	−1
tRNA-M	13561	13623	63	1

Table S1 (continued).

Genetic feature	Start	End	Length	Strand
tRNA-W	14573	14633	61	1
tRNA-C	14633	14690	58	−1
tRNA-V	11846	11897	52	−1
Control region	12624	13443	820	1
AT repeats	3955	4002	48	1
AT repeats	4639	4668	30	1
AT repeats	4646	4673	28	1
AT repeats	6486	6537	52	1
AT repeats	11475	11566	92	1
AT repeats	12706	12800	95	1
AT repeats	13106	13260	155	1
AT repeats	8630	8679	50	1
